# Computer Aided-Diagnosis of Prostate Cancer on Multiparametric MRI: A Technical Review of Current Research

**DOI:** 10.1155/2014/789561

**Published:** 2014-12-01

**Authors:** Shijun Wang, Karen Burtt, Baris Turkbey, Peter Choyke, Ronald M. Summers

**Affiliations:** ^1^Imaging Biomarkers and Computer-Aided Diagnosis Laboratory, Radiology and Imaging Sciences, Clinical Center, National Institutes of Health, Building 10, Room 1C224, Bethesda, MD 20892-1182, USA; ^2^Molecular Imaging Program, National Cancer Institute, National Institutes of Health, Building 10, Room B3B69F, Bethesda, MD 20892-1088, USA

## Abstract

Prostate cancer (PCa) is the most commonly diagnosed cancer among men in the United States. In this paper, we survey computer aided-diagnosis (CADx) systems that use multiparametric magnetic resonance imaging (MP-MRI) for detection and diagnosis of prostate cancer. We review and list mainstream techniques that are commonly utilized in image segmentation, registration, feature extraction, and classification. The performances of 15 state-of-the-art prostate CADx systems are compared through the area under their receiver operating characteristic curves (AUC). Challenges and potential directions to further the research of prostate CADx are discussed in this paper. Further improvements should be investigated to make prostate CADx systems useful in clinical practice.

## 1. Introduction

Twenty-eight percent of cancers in men occur in the prostate, making prostate cancer (PCa) and its detection a priority in cancer research [1]. Approximately 16% of men will be diagnosed with PCa within their lifetime [1]. Despite a steady and consistent increase in five-year survival rates from 66.0% (1975) to 99.6% (2005), PCa remains a major healthcare problem in the United States [2].

The reduction in mortality is widely attributed to early cancer detection and improvements in treatment. Because digital rectal examination (DRE) is only effective for identifying posterior peripheral zone tumors, it failed to detect many tumors that originated in the anterior peripheral zone, central zone, and transitional zones [3] as well as tumors that were too small to be palpated.

Prostate-specific antigen (PSA) testing became a common method of screening in the early 1990s and has since proven to be very controversial. Several large PSA screening trials have shown reduction in the risk of death due to PCa [4–6]. However, other large studies found conflicting results, reporting that PSA screening had no significant effect on PCa death rate [7, 8]. The United States Preventive Services Task Force (USPSTF) awarded PSA screening the letter grade of “D.” This has resulted in a trend away from its use in the USA.

Random systematic (sextant) biopsies under transrectal ultrasound (TRUS) guidance of the prostate are prone to discovering small, low grade cancers that may ultimately lead to treatment even though they are unlikely to result in death. Moreover, the random biopsy method is prone to low sensitivity in detecting clinically significant tumors [9–12]. Although TRUS is more convenient than MP-MRI and has a lower cost, its low sensitivity makes it unsuitable for screening a large patient population [13].

As a result of these limits, endorectal magnetic resonance (MR) imaging contributes significant value to PSA, DRE, and biopsy findings by localizing cancer and by assessing its size and extension [14, 15]. The ROC curve for localization of cancer is higher for endorectal imaging than for DRE in the apex, midgland, and base. Likewise, endorectal MR imaging is more accurate than TRUS-guided biopsy in tumor localization in the midgland and base of the prostate [16]. However, MRI is insensitive to whether the cancer has metastasized to the lymph nodes and is only somewhat accurate in predicting if the cancer has penetrated the prostate capsule [17]. A number of structures and conditions—including BPH nodules, prostatitis, and hemorrhage—show similar enhancement patterns to PCa on dynamic contrast-enhanced (DCE) MR images and therefore compromise the specificity of image analysis [18].

## 2. Clinical Advances in Multiparametric MRI for Prostate Cancer Diagnosis

Multiparametric (MP) magnetic resonance imaging (MRI) may improve the diagnosis and the care of PCa patients by providing morphological and functional information about the prostate. MRI sequences shown to correlate with properties associated with PCa include T2-weighted imaging (T2WI), diffusion-weighted imaging (DWI), MR spectroscopy, and dynamic contrast-enhanced (DCE) MRI [19–25]. MP-MRI is especially effective in revealing anterior prostate cancer in men with negative random transrectal 12-core biopsy [26]. In such cases, MP-MRI is beneficial for selecting men who should undergo anterior biopsy. Such an approach increases the positive predictive power of PCa diagnosis [27]. It is therefore highly recommended that MP-MRI is used rather than a single MRI modality when assessing a patient for prostate lesions.

By fusing endorectal coil MR images to a preprocedure TRUS using electromagnetic needle tracking, biopsies may be directed to suspicious lesions and the location of biopsies may be documented [28]. Targeted prostate biopsy with MP-MRI guidance has been shown to improve the sensitivity of prostate cancer detection when compared with random biopsy [29, 30]. MP-MRI/ultrasound image fusion reduces the number of required biopsies while also reducing the diagnosis of clinically insignificant cancer [31]. Initially, endorectal coils were used during MP-MRI to increase the signal-to-noise ratio (SNR). However, as MP-MRI has become more widespread and technology has improved, endorectal coils are no longer consistently used and the costs associated with them are avoided [32]. Futterer et al. found AUC to be significantly higher when endorectal coils were used when compared with pelvic coils [33]. Similarly, Turkbey et al. found that more cancer foci were detected using dual-coil prostate MRI than when nonendorectal coil MRI was used at 3T [34]. However, Bratan et al. contends this claim with findings that the field strength and the type of imaging coils used have no significant influence on the detection rate of tumors [35]. As technology evolves it is likely that there will be a decreasing need for endorectal coils.

## 3. Computer Aided-Diagnosis for Prostate Cancer

Interpreting MRI requires a high level of expertise and is time consuming. Significant interobserver variation and a lack of sensitivity, specificity, and accuracy exist for radiologists in interpreting the volume and stage of lesions in prostate MRI [36–38].

**Figure 1:**
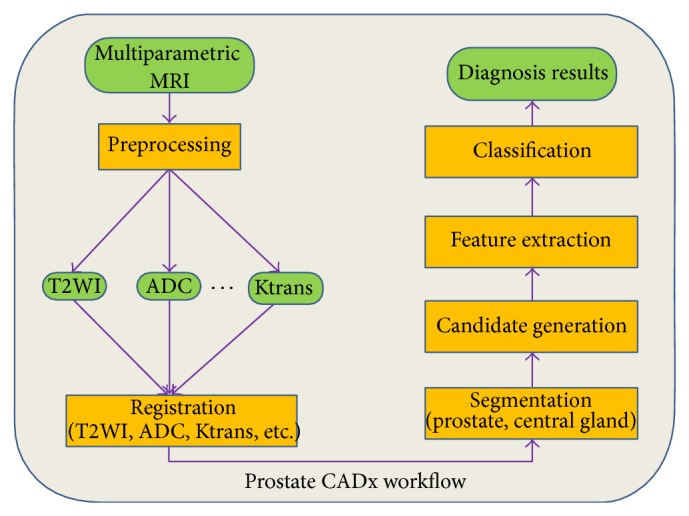
Workflow of a typical prostate CADx system. Green rectangles indicate data (original scans and images after preprocessing); yellow rectangles indicate processes applied to the data or images.

There is demand for an accurate computer aided-diagnosis (CADx) system that decreases reading time, reduces required expertise in radiology reading, and offers a consistent risk assessment of cancer presence in prostate MRI. Such a CADx system could automatically detect suspicious lesions in prostate MR images to help screen for prostate cancer in large patient populations. A typical CADx system for prostate cancer detection takes multiparametric MR images (MP-MRI), processes them, and generates a specific diagnostic result (e.g., a prediction map of the prostate showing regions with high probability to be cancer). There are some common components which are shared by prostate CADx systems such as feature extraction and classification. The workflow of a typical CADx system is shown in Figure 1.

### 3.1. Image Preprocessing

In the preprocessing step, raw data are processed to normalize the image or to transform the image to a domain where cancer can be easily detected. Challenges include variation between patients of intensity values on T2WI and the presence of a nonuniform multiplicative bias field within scans. Acquisition setup may also differ between patients. To reduce interpatient variance and to make the signal intensity consistent across the whole population, images are typically normalized. A standard method for normalizing T2WI images involves dividing the original intensity by median + 2 × (interquartile range) [39]. In the work of Shah et al., the authors normalized T2WI images using the average fat signal near the prostate [40]. DWI images are commonly converted to ADC maps [40, 41], which show better representation of lesions than DWI. The ADC map depicts a quantitative measure of the degree of molecular mobility. It is computed at the voxel level using the following function [40]: *S* = *S*_0_ × exp⁡(−*b* × ADC), where *S*_0_ is the pixel value at *b* = 0 and *b* is the diffusion gradient factor. Cancers restrict water motion (lower ADC value) due to the low permeability of their cell membranes compared to normal tissue where water motion is relatively higher (high ADC value).

### 3.2. Segmentation

The prostate must be segmented from the image prior to further analysis. An accurate prostate segmentation may assist in guiding radiotherapy, biopsy, and focal therapy in addition to its application in diagnosis. Because T2WI shows more detailed anatomical structures of the prostate than other MR sequences, it is widely used for segmentation of the prostate. After registration, the prostate segmentation can be applied to other image sequences.

Segmenting the prostate from T2WI is a challenging problem. The boundary between the prostate and the surrounding tissues can be difficult to locate. Even for experienced radiologists, the interobserver variability of manual prostate segmentation is large. MR scans from different medical centers or institutes may have considerable differences based on the imaging protocols employed. For instance, endorectal coils induce deformations that cause scans to appear different than those using no such coil.

Atlas-based registration is a mainstream method for segmenting the prostate on MRI. Klein et al. proposed a segmentation method based on nonrigid registration of a set of 3D labeled atlas images using localized mutual information [42]. Martin et al. proposed a two-step approach for automated prostate segmentation [43]. In the first stage, a probabilistic prostate atlas was employed to estimate a rough localization of the prostate; in the second stage, a spatially constrained deformable model was refined toward the prostate boundary. To evaluate atlas-based automatic and semiautomatic segmentation strategies for prostate MRI, Martin et al. conducted a multiphase validation study which assessed both segmentation time and accuracy [44]. A Dice similarity coefficient, comparing the spatial overlap of voxels in two volumes, was reported as 0.94 for the autosegmented contours with pre- and postmanual edits. Using the N points strategy reduced segmentation time by 49% when compared with manual segmentation.

Deformable models segment the prostate through the influence of internal forces which smooth the boundary and external forces which move the model toward the object boundary. Chandra et al. proposed a fast segmentation method based on a case specific deformable model for MR prostate scans without an endorectal coil [45]. Yin et al. employed a two-step approach for fully automated and robust prostate segmentation: first, the prostate region is detected based on the cross correlation of normalized gradient fields; second, a prostate mean shape model is refined by means of a graph-search framework [46]. Deformable models are useful when noise and sampling artifacts result in invalid object boundaries.

The graph-cut algorithm may be used to find a globally optimal solution to a segmentation problem. Mahapatra and Buhmann proposed a prostate MRI segmentation algorithm using learned semantic knowledge and graph cuts [47]. To identify volume of interest (VOI), they employed supervoxel segmentation and used random forest to estimate the location of the prostate. A second set of random forest classifiers was trained and applied based on image and context features to refine VOI probability at the voxel level. A Markov random field was built and optimized using graph cuts to get the final segmentation of the prostate. A Dice metric of 81.2 ± 4.5% indicates that using the graph-cut algorithm with semantic knowledge is an effective segmentation technique, although this value was lower than for equivalent segmentation systems that were cited.

To compare state-of-the-art prostate segmentation methods, a prostate segmentation challenge workshop was set up and hosted by the MICCAI 2012 conference [48]. This challenge provided a dataset of 100 prostate MR cases from 4 different centers, with differences in scanner manufacturer, field strength, and protocol. 11 teams with academic research or industry backgrounds participated in the challenge. The Imorphics and ScrAutoProstate teams achieved the highest overall scores of 85.72 and 84.29, respectively. The overall score is a mapping function which incorporates completely different but equally important metrics like Dice coefficient and average boundary distance, defined as the mean minimum distance between the manually segmented ground truth and automatically segmented boundary. Imorphics' algorithm is based on the active appearance model; ScrAutoProstate's algorithm is a region-specific hierarchical segmentation method using discriminative learning. Algorithms of both teams showed significantly better results when compared with other teams' algorithms. The final results, although promising, showed that the prostate segmentation problem is still unresolved.

### 3.3. Registration

Image registration methods have become an essential part of radiological imaging in the last decade. Because prostate cancer shows different characteristics on multiparametric MRI, ultrasound and whole mount histology, analyzing a fusion of all modalities, lead to a better diagnosis of prostate cancer. Patient movement during MRI results in translation and distortion of the prostate, which must be corrected for by registering MRI sequences to each other before feature extraction and classification. 3D images must be registered before information from different modalities/sequences is fused.

Currently, pathological analysis of prostatectomy specimens is the reference standard for determining the ground truth of prostate cancer. In order to transfer the labels from pathology to MP-MRI, MR images must be registered with pathological sections of the prostate. The nature of the pathological sections is quite different from that of the MRI sections. For instance, the MRI section is typically 3 mm thick whereas the pathologic section represents a 5 *μ*m thick subsample of the larger 3–5 mm thick slice. This contributes to the difficulty of designing accurate registration algorithms. Mazaheri et al. proposed a free-form deformation algorithm based on B-splines for registration of prostate MR images to pathological slices [49]. As a result of registration, Dice values increased from 0.86, 0.65, and 0.89 to 0.99, 0.89, and 0.97 for the WP, PZ, and TZ, respectively. While this method was successful for in-slice registration of the T2WI image with the pathological slice, slices had to be matched manually.

To register T2WI, ADC, DCE, and whole mount histology (WMH) images, Chappelow et al. proposed an automated elastic registration method utilizing a multivariate formulation of mutual information of data from all modalities [50]. Their technique improved the accuracy of registering in vivo MP-MRI and ex vivo WMH of the prostate when compared to prior approaches based on mutual information.

In the work of Liu et al., T2WI, ADC images, and Ktrans maps were registered using coordinate information stored in the DICOM image headers [39]. Each image slice was considered as a plane with an origin and orientation given in the header information. To match corresponding voxels, voxels in the most highly resolved series (T2WI) were projected onto the closest slice of the other imaging modalities. This is a simple registration method which is effective when deformation of the prostate is minor enough to not warrant a registration algorithm.

The optimal registration method for MR images depends largely on the imaging protocol. A simple registration method, such as that used in Liu et al. [39], is often adequate when patient motion is minimal. However, uncomfortable protocols (i.e., endorectal coil use) or protocols with a long time frame (i.e., DCE imaging) increase the likelihood that considerable patient movement will occur and result in the translation of the prostate. This necessitates the use of more advanced registration techniques.

### 3.4. Feature Extraction

Extracting distinctive features from targets of interest is a key characteristic of a successful CADx system. Typical features for medical images include volume, shape, texture, intensity, and various statistics. Many advanced classification techniques have been developed for machine learning. In theory, support vector machines (SVMs) could achieve the highest performance (global optimal) based on the maximization of the margin between positive and negative training samples. In practice, however, choosing which features are fed into the classifier is more important than choosing the classifier itself.

Designing an effective image feature set plays an important role in a CADx system. Because ADC maps detect prostate cancer better than other multiparametric MR images, research in the use of these maps is on the rise. Peng et al. studied ADC maps and used the 10th percentile and average ADC values as features [51]. Experimental results showed that when these features were combined with the T2WI signal intensity histogram skewness and the Tofts *K* ^trans⁡^ map, the CAD system achieved an AUC of  0.95 ± 0.02 in the differentiation of prostate cancer from normal foci. This outcome is currently the highest performance reported in the literature.

Diffusion tensor MR imaging (DTI) is a useful tool for prostate cancer detection. DWI and DTI characterize the dephasing of the MR signal as it relates to molecular diffusion. Pathological changes such as increases in cellular density caused by the prostate cancer can decrease the signal intensity in ADC and the average diffusivity 〈*D*〉 values in DTI. In DTI, structural changes caused by the prostate cancer can be shown by fractional anisotropy (FA). Moradi et al. conducted research on the detection and grading of dominant prostate tumors using DCE and DTI scans [52]. They employed 5 features: *K* ^trans⁡^, *v*_*e*_ and *v*_*p*_ extracted from DCE, and 〈*D*〉 and FA extracted from DTI. An AUC of 0.96 for this work indicates that DTI may offer information with high diagnostic quality for feature selection. However, in their experiment, ROIs were selected manually which may bias the performance. Systems relying on manual ROIs or biopsy locations for candidate generation are semiautomatic CADx systems. Candidate generation, or the process of identifying potentially suspicious regions for analysis, must be accomplished algorithmically for the CADx system to be considered fully automated. Developing a fully automatic CADx system with minimal radiologist intervention is a key factor in the successful deployment of a prostate CADx system.

When feature selection and dimensionality reduction is utilized, classification performance improves as more features are extracted from lesion candidates. In the work of Niaf et al., the authors extensively studied feature extraction for PZ cancer diagnosis on T2WI, DWI, and DCE images [53]. 140 features were extracted from lesion candidates on MP-MR images. These features were split into two groups: image features and functional features. Image features included grey-level, texture, and gradient features. Intensity values of three MP-MR images were directly used as grey-level features. First-order texture features—mean, median, standard deviation, and average deviation—were computed from a local 9 × 9 window. 19 second-order texture features were derived from the grey-level cooccurrence matrix (GLCM). These texture features characterized homogeneity, grey-level transitions, and anatomical structures. Gradient features were computed by using three gradient operators: Sobel filter, Kirsch filter, and a numerical gradient. Function features were solely extracted from DCE sequences and included semiquantitative and quantitative features. Semiquantitative features including wash-in (WI) and wash-out (WO) rate, SI peak (absolute maximum enhancement), SI max (95% of maximum enhancement), onset time, time-to-peak, time to max (Tmax), and the area under the gadolinium curve (AUGC) were derived from the enhancement curves. Quantitative features were computed based on a kinetic model of the enhancement curve. These features included the forward volume transfer constant (*K*_trans⁡_), the fractional volume of extracellular space per unit volume of tissue (*v*_*e*_), and the reverse reflux rate constant between extracellular space and plasma (*k*_ep_). The arterial input function (AIF) was measured by using regions of interest (ROI) drawn in the common femoral artery.

Vos et al. extracted first-order statistics such as the 25% and 75% percentiles from ROIs on Ktrans maps and T1 images [54]. Accuracy of 0.83 (c.i. [0.75–0.92]) was reported for discriminating malignant lesions from nonmalignant suspicious enhancing areas located in the normal PZ of the prostate. Similarly, Artan et al. utilized only intensities of T2, ADC, and *k*_ep_ images for prostate cancer localization [55]. The proposed system achieved 79% AUC, which was a decent percentage considering that only basic features were utilized.

Chan et al. employed two types of second-order features: texture features and anatomical features [56]. In their research, texture features were extracted from lesion candidates by using a cooccurrence matrix (CM) and a discrete cosine transform (DCT). The anatomical location of voxels was described by a cylindrical coordinate system. Litjens et al. used intensity, texture, shape (blobness), anatomy, and pharmacokinetic features on T2, proton-density weighted, diffusion weighted, and DCE images [57]. The peripheral zone probability feature determined whether a voxel belonged to the peripheral zone or the central gland using a pattern recognition framework that utilized texture, intensity, and anatomical features. DCE images were analyzed by fitting a biexponential curve to the time data; the parameters tau and LateWash, corresponding to time-to-peak and the slope of the last portion of the enhancement curve, respectively, were incorporated as features. These features were selected based on PI-RADS guidelines for reading prostate MR images. Using second-order features contributed to the ability of this system to achieve an equivalent performance to radiologists.

Another approach to feature extraction utilizes fractal geometry, which is capable of characterizing heterogeneities within an image. To detect prostate cancer on T2WI, Lopes et al. employed fractal and multifractal features to analyze textural properties of images [58]. These two types of features were computed using the variance method and the multifractional Brownian motion model, respectively. The use of fractal and multifractal features improved classification accuracy and reduced the influence of signal intensity variations when compared to textural features.

Wavelet transformations are widely used in signal processing for noise reduction, compression, digital encryption (e.g. digital watermark), and reconstruction [59–62]. Because they have applications in extracting representative signatures from multifrequency channels at various resolutions, wavelet transformations are also used in image analysis for feature extraction. Tiwari et al. proposed a multimodal wavelet embedding representation for feature extraction from MP-MRI [63]. The authors extracted 171 Haar wavelet features from magnetic resonance spectroscopy (MRS) and 54 Gabor features from T2WI. They then applied dimensionality reduction to each of the two groups of wavelet features and projected them to a common reduced eigenvector space. Tiwari et al. showed that the wavelet embedding system produced the most accurate prediction and highest AUC when compared with the T2WI or MRS feature vectors alone and other state-of-the-art combination-of-data systems.

The central gland (CG) and the peripheral zone (PZ) are used as anatomical coordinates in prostate biopsies to report the location of a cancer. Because cancer shows different characteristics in different regions of the prostate, incorporating anatomical structural information in a CADx system may improve the performance. Manual segmentation of the PZ and CG is time consuming; so it would be ideal if anatomical features could be extracted from ROIs. Liu and Yetik proposed a new feature called the location map, which is constructed by applying a nonlinear transformation to the spatial position coordinates of each pixel [64]. The location map could differentiate the transition zone (TZ) and PZ. Experimental results show that the detection sensitivity was improved when the new anatomical feature was combined with other nonanatomical features.

The work described above shows that while statistical features (i.e., Intensity features, histogram of gradients, etc.) have been commonly included in classification systems, features which capture geometric information have been less widely used. This trend would indicate that while work on statistical features is comprehensive and sufficient, the inclusion of features describing shape and symmetry within the prostate would greatly contribute to advancing the field of feature selection and extraction as it applies to machine learning.

### 3.5. Classification

The final step of a CADx system involves training and testing with features extracted from images and labels. A classifier is usually trained based on the labeled training set and applied to test cases without knowledge of true labels.

**Figure 2:**
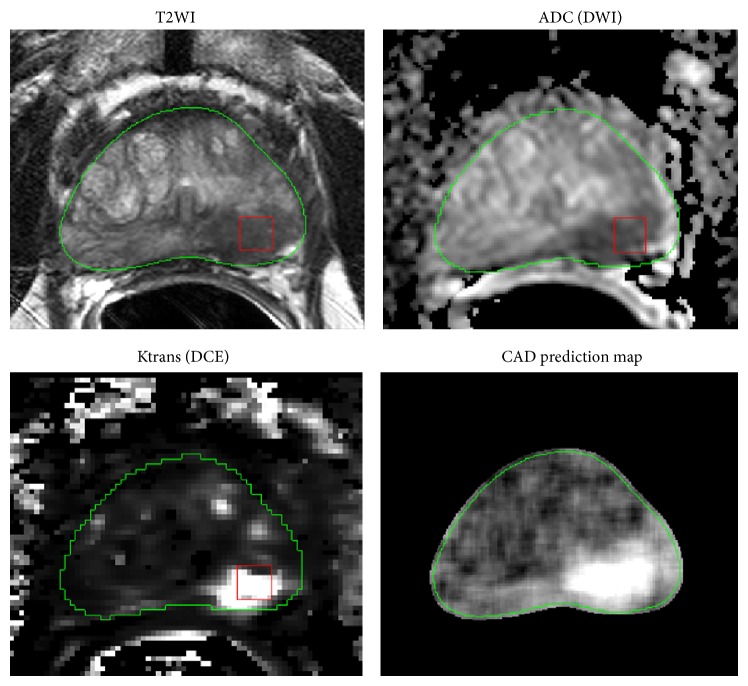
Illustration of a prostate CAD prediction map showing true positive cancer classified correctly by SVM. The red rectangle indicates an image patch within the cancer in which local image features are extracted from T2WI, ADC, and Ktrans map. The green contour denotes the boundary of the prostate. Bright regions in the CAD prediction map correspond to a high probability of cancer and coincide with the correct location of the cancer.

In the past two decades, SVMs have shown their effectiveness on many real-life classification problems [65]. The strong generalization ability of SVMs comes from the margin-maximization criterion. Vos et al. employed SVM for analyzing prostate lesions in the PZ using DCE MRI [54]. Later, Vos et al. applied SVM to the same problem using T2WI and DCE MRI [66]. Traditional SVMs treat errors with uniform cost. For prostate cancer diagnosis, detecting false negatives is vital as their cost to patient health is much higher than the cost of false positives. In the work of Artan et al., the authors applied the cost-sensitive SVM to prostate cancer localization and compared it with classical SVM [55]. Moreover, they combined conditional random fields (CRF) with a cost-sensitive framework for segmenting the lesion and found that this method improved cost-sensitive SVM results by incorporating spatial information. Liu and Yetik fed SVM with a location map and with multiparametric MR images to segment prostate cancer [64]. They found that it is feasible to detect tumors without PZ extraction by fusing the spatial map. Shah et al. employed SVM to localize prostate cancer based on multiparametric MRI [40]. To find optimal hyperparameters for SVM, they used a genetic algorithm in which each SVM hyperparameter was treated as a “gene” in a “chromosome” encoding. In this evolutionary strategy approach, the *F*-measure was used as the metric for the evaluation of individual fitness. Chan et al. compared a single-channel maximum likelihood classifier based solely on image intensities to a support vector machine (SVM) and Fisher linear discriminant (FLD) which utilized five different sets of derived features [56]. FLD showed the highest performance when compared with the other two classifiers. In a later study [53], Niaf et al. compared the performance of SVM, linear discriminant analysis (LDA), a Naïve Bayes classifier, and the k-nearest neighbors algorithm on the diagnosis of prostate cancer in the peripheral zone using T2WI, DWI, and DCE images. SVM achieved the highest performance in this study. LDA was also employed by Peng et al. in the development of texture features on T2WI and ADC features on DWI [51, 67]. Other works utilizing SVM for classifying prostate cancer on multiparametric MRI include Liu et al. and Moradi et al. [39, 52]. In Figure 2, we show an exemplar prediction map generated by a prostate CAD system developed by Liu et al. [39].

In recent years, random forests were introduced to the area of medical image analysis and achieved very promising results in some medical applications [68]. Random forests are an ensemble learning classification method [69, 70]. They build decision trees with random perturbations of training samples and features to ensure high generalization ability. Random forest is one type of random subspace learning method which builds an ensemble of classifiers by exploring partial feature spaces (also called subspaces) of the input data [69]. Tiwari et al. employed random forests in the task of prostate cancer classification based on the combined eigenvector representation of T2WI, MRI, and MRS channels [63]. Experimental results showed that random forest has a strong capability for integrating any combination of heterogeneous data modalities with various scales and dimensions.

Since kernel-based learning methods (e.g., SVMs) are commonly used to classify prostate cancer on multiparametric MRI, developing more specific kernel-based classifiers and adapting them to the prostate MRI domain may lead to further improvement of prostate CADx systems. In the CADx system developed by Tiwari et al., there are three main modules: multikernel learning, semisupervised learning, and dimensionality reduction [71]. Experimental results showed that this elaborately developed kernel-based learning system is a powerful diagnostic and prognostic tool for prostate cancer diagnosis on MRI/MRS.

In the work discussed in this section, classification was achieved automatically by employing different forms of statistical classifiers. To simulate the diagnostic process of radiologists, Puech et al. proposed a semiquantitative rule-based classification method for prostate cancer diagnosis on DCE MRI [72]. In this method, a scoring algorithm was designed based on the wash-out slope (Wo), maximum wash-out rate (Max Wo PCa), minimum wash-in rate (Min Wi PCa), median wash-out rate (Med Wo PCa), and median wash-in rate (Med Wi PCa) in the area of the lesion as measured on DCE MR images. This method is unique in that it directly encodes reading experience of radiologists without resorting to a statistical classifier to implicitly extract classification rules from labeled data.

With well-registered images and informative feature extraction, prior work indicates that SVM and random forest work well on the problem of classifying prostate tumors on MP-MRI.

## 4. Performance Comparison

Table 1 compares the performance of the major published prostate CADx systems. Some papers investigated several techniques for prostate cancer diagnosis. For such papers, only the highest performance and the corresponding classifier are listed. For candidate generation, “voxel” indicates that each voxel from the image was treated as one lesion candidate. Due to various factors involved such as patient population, data size, modality, and region of interest, the comparisons shown below may not be fair in regard to performance. However, Table 1 still gives a sense of how well a CADx system is capable of analyzing prostate cancer on MRI.

## 5. Discussion and Perspectives

Table 1 shows several consensuses in the field of prostate MRI CADx research. A combination of T2WI, DWI (ADC), and DCE MRI is the most commonly used set of parameters for prostate cancer imaging. Additionally, histological interpretations from in vivo or ex vivo biopsy specimens were widely used to determine ground truth on MP-MRI. In vivo biopsy can only label the pathology of points inside the prostate. Radiologists must manually define lesion boundaries on MP-MRI retrospectively based on the biopsy results. Ex vivo whole mount prostate histological analysis provides more accurate label information for training a CADx system. However, whole mount histology is expensive and registering whole mount histological slices with 3D MP-MRI is a challenging problem. Moreover, research focus has shifted from PZ prostate cancer to whole prostate cancer diagnosis in recent years, despite this approach being more challenging than PZ lesion analysis. Kernel-based learning methods such as SVMs showed high sensitivity and specificity and were employed by a majority of research groups for classifying prostate cancer from normal tissue. While CADx systems tested on images from machines using both 1.5T and 3T field strengths showed similar performances, the two systems with the best performance both used images obtained at 3T. This may be in part because of an improved signal-to-noise ratio resulting in greater resolution of the images. Regarding data size, the majority of prostate CADx systems employed a relatively small data set with no more than 50 patients. Validation on a large-scale data set with several hundred patients is required to make the systems usable in clinical settings.

There are challenges facing the field of prostate CADx research. The majority of the prostate CADx systems listed in Table 1 reported AUC in the range from 0.80 to 0.89. Moradi et al. and Peng et al. reported AUCs of 0.96 and 0.95, respectively, which represent the highest performance in the group [51, 52]. However, their systems generated lesion candidates based on biopsy locations or manually selected ROIs which may be data set dependent. The generalization of this system to other unseen test sets needs to be validated.

Reducing the false positive rate is vital to improving the performance of a prostate CADx system. In Liu et al. [39], many false positives were from benign prostatic hyperplasia (BPH) nodules. BPH nodules show similar enhancement patterns to prostate cancer on MP-MRI. Like PCa, BPH nodules also show early enhancement at wash-in phase and their intensity drops more quickly at wash-out phase than normal prostate tissue on a DCE sequence. Detecting BPH nodules on MP-MRI is a key factor in reducing false positives for a prostate CADx system.

While the MP-MRI combination of T2WI, DWI, and DCE images is becoming mainstream for prostate CADx systems, the exploration of image modalities such as MRS and DT has also been observed. The introduction of new imaging modalities or new modality combinations for MP-MRI may lead to better CADx systems. Because images or signals are the information source of all CADx systems, the development and adoption of more accurate scanning methods will alleviate the burden imposed on the postprocessing CADx systems.

To compare state-of-the-art CADx systems for prostate cancer diagnosis on MP-MRI, a publicly available dataset is necessary to provide a fair comparison for different techniques. The public dataset should include major MRI modalities such as T2WI, DWI, and DCE from various MRI vendors. The patient population in the data set should be large enough to include anatomical and pathological patient variations. To develop supervised learning techniques for prostate cancer diagnosis, it is necessary that accurate labels of the cancer exist for MP-MRI images. Such labels should be confirmed by histological analysis. Ideally, these labels or cancer contours should be registered to the whole mount prostate histology images.

In the field of prostate segmentation for MRI, we are pleased to see the PROMISE12 challenge has been successfully organized [48]. Challenges and publicly available data sets such as PROMISE12 support the advancement of prostate segmentation for MRI though motivation and consistency.

## 6. Conclusion

In this paper, we surveyed computer aided-diagnosis systems for detection and diagnosis of prostate cancer on MP-MRI. From the survey it was determined that current major CADx systems recorded AUC performance below 0.90. This finding indicates that there is still room for improvement through future research in this field. Prostate cancer CADx systems are a complicated composition of image normalization, preprocessing, segmentation, registration, feature extraction, and classification modules. It is therefore expected that development and progress in one or more of these modules should lead to a better diagnostic system with higher sensitivity and lower false positive rate, as has been observed from previous research efforts. It is likely that more improvements will be made in the next decade and wide deployment of prostate CADx systems in the clinical environment will eventually occur.

## Figures and Tables

**Table 1:** Performance comparison of major prostate CADx systems published.

Publication	Data size	Modality	Field strength	Ground truth	Candidate generation	Region	Classifier	Performance
Chan et al. 2003 [56]	15	T2WI, ADC, PD, and T2 map	1.5T	MSTR + biopsy	RS + cancer	PZ	FLD	AUC = 0.839 (±0.064)
Puech et al. 2009 [72]	100	DCE	1.5T	Biopsy + RS	Manual ROI	WP	Rule-based	AUC = 0.77
Vos et al. 2008 [54]	34	DCE	1.5T	Biopsy	Manual ROI	PZ	SVM	AUC = 0.83, CI [0.75, 0.92]
Vos et al. 2010 [66]	34	T2WI, DCE	1.5T	Biopsy	Manual ROI	PZ	SVM	AUC = 0.89, CI [0.81, 0.95]
Shah et al. 2012 [40]	31	T2WI, ADC, and DCE	3T	Biopsy	Manual ROI	PZ	SVM	*F*-measure = 0.89
Liu and Yetik 2011 [64]	20	T2WI, ADC, and DCE	1.5T	Biopsy	Voxel	WP	SVM	AUC = 0.89
Liu et al. 2013 [39]	54	T2WI, ADC, and DCE	3T	Biopsy	Biopsy spots	WP	SVM	AUC = 0.82, CI [0.71, 0.93]
Niaf et al. 2012 [53]	30	T2WI, ADC, and DCE	1.5T	MSTR + biopsy	Manual ROI	PZ	SVM	AUC = 0.89, CI [0.81, 0.94]
Moradi et al. 2012 [52]	29	DT, DCE	3T	Biopsy	Biopsy spots	WP	SVM	AUC = 0.96
Niaf et al. 2014 [73]	49	T2WI, ADC, and DCE	Not specified	MSTR + biopsy	Manual ROI	WP	P-SVM	AUC = 0.889
Peng et al. 2013 [51]	48	T2WI, ADC, and DCE	3T	MSTR + biopsy	Manual ROI	WP	LDA	AUC = 0.95 (±0.02)
Artan et al. 2010 [55]	21	T2WI, ADC, and DCE	1.5T	Biopsy	Voxel	PZ	CRF	AUC = 0.79 (±0.12)
Tiwari et al. 2013 [71]	29	T2WI, MRS	1.5T	MSTR + biopsy	Voxel	WP	SeSMiK-GE + random forest	AUC = 0.89 (±0.09)
Tiwari et al. 2012 [63]	36	T2WI, MRS	1.5T	MSTR + biopsy	Voxel	WP	Random forest	AUC = 0.89 (±0.02)
Litjens et al. 2014 [57]	347	T2WI, PDWI, DCE, and DWI	3T	Biopsy	Voxel	WP	Random forest	AUC = 0.889

PD: proton density; MSTR: manual segmented tumor by radiologist; RS: random sampling; AUC: area under the ROC curve; HMM: hidden Markov models; WP: whole prostate; CI: confidence interval; ROI: region of interest; RG: region growing; FSE: fast spin-echo; DT: diffusion tensor; P-SVM: probabilistic SVM; SeSMiK-GE: semisupervised multikernel graph-embedded, PDWI: proton density-weighted imaging.
